# Neonatal diagnosis of Marcus Gunn jaw‐winking syndrome

**DOI:** 10.1002/ccr3.3664

**Published:** 2020-12-20

**Authors:** Daniela David, Valentina Chiavaroli, Modesto Lanci, Laura Sabatini, Silvia Greco, Silvia Carinci, Marianna Sebastiani, Eleonora Coclite, Francesco Chiarelli, Susanna Di Valerio

**Affiliations:** ^1^ Department of Pediatrics Gabriele d'Annunzio University of Chieti and Pescara Chieti Italy; ^2^ Neonatal Intensive Care Unit Pescara Public Hospital Pescara Italy; ^3^ Liggins Institute The University of Auckland Auckland New Zealand; ^4^ Department of Ophthalmology Pescara Public Hospital Pescara Italy

**Keywords:** Marcus Gunn syndrome | newborn | ophthalmology; congenital ptosis

## Abstract

This report highlights the importance for neonatologists/pediatricians of considering Marcus Gunn jaw‐winking syndrome among differential diagnoses of ptosis. A detailed clinical assessment is crucial to promptly recognize and appropriately manage it.

## Graphical abstract

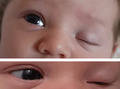

## 1 INTRODUCTION

Left‐eye ptosis was observed in a newborn soon after birth. At the 15‐day follow‐up ophthalmic visit, no improvements were observed. Namely, abnormal left eyelid cooperation and uncoordinated opening/closing movements with paradoxical synkinesis in swallowing were noted. These clinical findings have allowed diagnosing Marcus Gunn jaw‐winking syndrome.

Marcus Gunn jaw‐winking syndrome is a type of neurogenic congenital ptosis that includes an associated winking motion of the affected eyelid each time the jaw moves.^1^, ^2^ Elevation and even retraction of the affected eyelid can be triggered by several mouth movements, such as chewing, suction, protruding tongue, or smiling.^1^, ^3^ This syndrome is likely to occur from an abnormal branch of the trigeminal nerve that, for congenital misdirection, supplies the levator palpebrae superioris muscle of the oculomotor nerve.^1^ It has been observed in 2%‐13% of patients with congenital ptosis, with equal prevalence in males and females.^1^

Here, we report the case of a male newborn with Marcus Gunn jaw‐winking syndrome involving the left eye, which was diagnosed at 2 weeks of age.

## 2 CASE HISTORY/EXAMINATION

This male newborn was delivered at 39 weeks' gestational age by Cesarean section due to a failed trial of labor. At delivery, the Apgar score was 8 at 1 minute and 10 at 5 minutes of life. Birth anthropometry taken was as follows: weight 3560 gr (75th percentile), length 51 cm (75th percentile), and head circumference 33 cm (10th percentile). The boy was the first‐offspring of unrelated healthy parents. Family history was negative for congenital malformations and disorders. Pregnancy was unremarkable.

**FIGURE 1:**
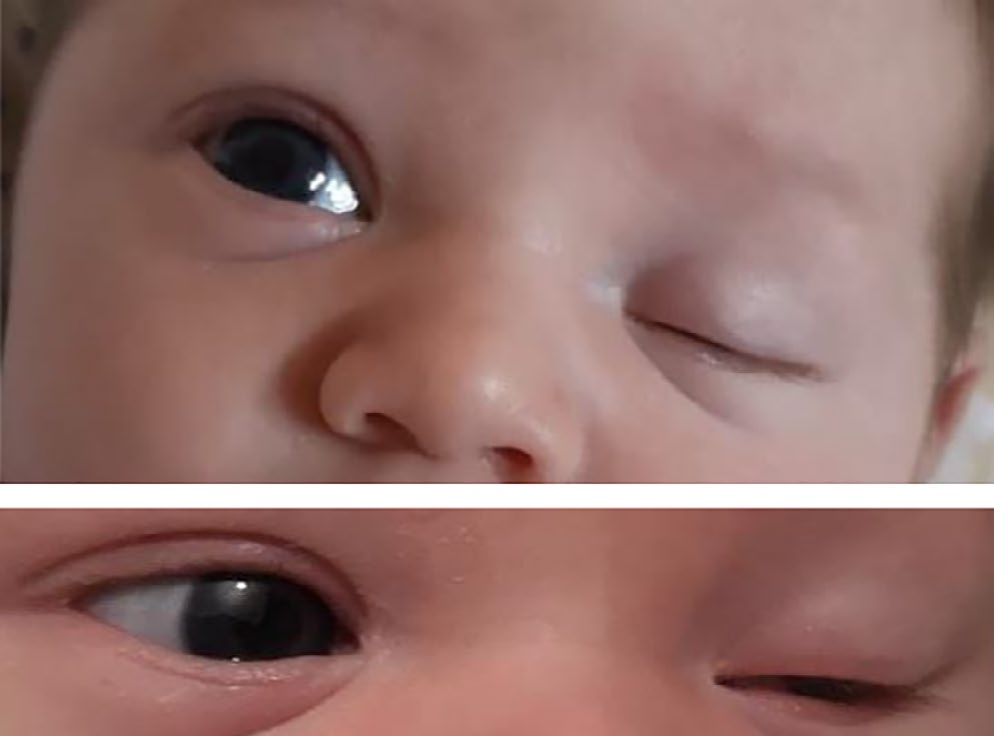
Left‐sided ptosis with the newborn hardly able to lift his left eyelid, whose opening was below the upper eyelid margin

At the initial full clinical examination performed soon after birth, left‐eye ptosis was observed without evidence of edema. Namely, the newborn was hardly able to lift his left eyelid, whose opening was below the upper eyelid margin (Figure 1). Pupils were bilaterally isochoric, isocyclic, and reactive to light. Newborn's general appearance and clinical conditions were otherwise normal. No nervous system anomalies were detected. At 3 days of life, a first ophthalmologist evaluation was performed, and possible congenital ptosis was suspected. The red reflex test was normal. Brain sonography excluded abnormalities. The newborn was discharged at 4 days of life in good clinical conditions, with a follow‐up program of the ptosis.

### 2.1 Differential diagnosis, investigations, and treatment

At the 15‐day follow‐up ophthalmic visit, no improvements of the left‐eye ptosis were detected. Since the baby was delivered by an uneventful Cesarean section, a traumatic nature of the left‐eye ptosis was excluded. Instead, the suspect of congenital ptosis was confirmed.

Differential diagnosis of congenital ptosis included (a) anomalies of extraocular muscle development and (b) anomalies of innervation. The first group comprises several disorders, such as Marcus Gunn jaw‐winking syndrome, inverse Marcus Gunn phenomenon, and Duane's syndrome.^4^ Congenital ptosis due to anomalies of innervation may be the result of: (a) neurologic dysfunctions; (b) neuromuscular junction failure of the levator muscle; or (c) dysfunction of the sympathetic nervous system (eg, Horner syndrome).^5^

In the present case, at the follow‐up ophthalmic visit the detection of abnormal left eyelid cooperation with uncoordinated opening/closing movements was noted, together with paradoxical synkinesis in swallowing during bottle‐feeding (Videos S1 and S2). Based on these peculiar features, Marcus Gunn jaw‐winking syndrome was diagnosed. Conversely, the inverse Marcus Gunn phenomenon was excluded. Indeed, in this rare congenital disorder, mouth opening (eg, while the individual is eating) induces or worsens eyelid drooping, due to an anomalous connection between the oculomotor and trigeminal nerves.^4^ Similarly, Marin‐Amat syndrome, an acquired type of oculofacial synkinesis, was excluded as this rare condition is characterized by involuntary and fleeting eye closure when the mouth opens (*eg,* while the individual is smiling), due to an anomalous connection between the trigeminal and facial nerves.^4^, ^6^ Lastly, Duane's syndrome, also known as Duane retraction syndrome, was not suspected because this ocular disorder is characterized by retraction movements and palpebral fissure narrowing when adduction is attempted, sometimes associated with downshoot and upshoot, and varying degrees of horizontal duction deficiency.^7^

### 2.2 Outcome and follow‐up

At the 4‐month follow‐up ophthalmic visit, no substantial improvements in the left‐eye ptosis were detected. Physical growth and development were otherwise normal. A further follow‐up visit was organized at the age of 8 months to assess the need and timing of corrective surgery.

## 3 DISCUSSION

Marcus Gunn jaw‐winking syndrome has been recognized as a distinct clinical entity for more than a century, after being first reported in 1883.^2^ This peculiar type of congenital ptosis is characterized by an upward jerking of the affected eyelid every time the jaw moves and is due to an anomalous neurological connection between the trigeminal and oculomotor nerves.^1^, ^2^ Rare familial cases with an irregular autosomal dominant inheritance pattern have been reported.^1^

The phenomenon of jaw‐winking ptosis can be bilateral, although it is almost always sporadic with the left side most commonly involved (as in our patient).^1^ It is generally diagnosed early in life, predominantly based on parental or other caregiver description of the synkinetic movement during the child's feeding. However, in some cases, this syndrome can go unnoticed until adolescence.^1^

Varying degrees of ptosis have been identified as follows: mild (≤2 mm), moderate (3 mm), or severe (≥4 mm).^4^ A variability of degrees of lid elevation has also been observed, given also by the upper eyelid motion, stimulated by the ipsilateral external pterygoid and the levator palpebrae muscles.^8^ The phenomenon of jaw‐winking ptosis tends to improve with age.^1^ However, improvements can only be apparent due to the patient's constant jaw contraction.^9^ Indeed, over time patients became aware of the trigger movements and learn how to avoid them and minimize/mask the syndrome.^10^, ^11^

Associated complications of Marcus Gunn jaw‐winking syndrome include strabismus and anisometropia (50%‐60% and 5%‐25% of cases, respectively).^12^ Amblyopia can occur in 30%‐60% of patients ^12^ and, in most cases, is secondary to strabismus or anisometropia; only rarely, it is due to occlusion by a ptotic eyelid.^1^ The coexistence of these complications needs to be investigated, especially in those individuals in whom the syndrome is diagnosed during adolescence, and the above‐mentioned visual defects may already be established. Of importance, any associated conditions, especially strabismus and amblyopia, should be corrected before contemplating surgical management of the jaw wink.

In the case of congenital ptosis of mild characteristics and without major refractive errors, the surgical repair should be performed only for cosmetic reasons and based on the psychological impact of the ptosis on child.^5^ Conversely, the most severe cases need necessarily a surgical approach, which is carried out through unilateral levator excision and frontalis brow suspension. To avoid asymmetry due to unilateral suspension, bilateral frontalis suspension has been recommended.^1^ Gene therapy may represent a novel approach to replace mutated genes with their healthy copy.^5^ In the present case, ophthalmic follow‐up was arranged at the age of 8 months to determine the need for corrective surgery.

In conclusion, this case report highlights the importance of considering Marcus Gunn jaw‐winking syndrome in the differential diagnosis of congenital ptosis. Indeed, neonatologists and pediatricians should be aware of this particular disorder in infants and children with signs of ptosis that improve in response to mouth movements (eg, sucking and swallowing). Thus, a detailed and prolonged clinical assessment plays a pivotal role in allowing a prompt diagnosis, so that this disorder can be explained to parents avoiding apprehension. Of importance, a prompt diagnosis helps in preventing the development of adaptive phenomena and ophthalmic disorders and allows to establish the appropriate management.

## CONFLICT OF INTEREST

None declared.

## AUTHOR CONTRIBUTIONS

DD and VC: wrote the manuscript. VC, LS, SC, and MS: examined the patient. ML: made the diagnosis. SG and EC: were involved in the literature search and drafting of the paper. FC and SDV: coordinated and approved the final version of the manuscript.

The content has not been published or submitted for publication elsewhere.

## ETHICAL APPROVAL

Verbal and written consent was obtained from the parents regarding the publication of the case and images. This report does not contain any personal information that could lead to the identification of the patient.

## Supporting information

Video S1

Video S2

## Data Availability

Data sharing was not applicable—no new data were generated.
